# Embodiment and Psychological Health in Adolescence: 1. Development and Validation of a Brief 12-item Questionnaireto Measure the Experience of Embodiment

**DOI:** 10.17505/jpor.2025.27576

**Published:** 2025-04-01

**Authors:** Lo Foster, Lars-Gunnar Lundh, Daiva Daukantaitė

**Affiliations:** 1 Department of Psychology, Lund University, Lund, Sweden, lo.foster@psy.lu.se ORCID: https://orcid.org/0009-0008-3636-956X; 2 Department of Psychology, Lund University, Lund, Sweden, lars-gunnar.lundh@psy.lu.se ORCID: https://orcid.org/0000-0002-1649-969X; 3 Department of Psychology, Lund University, Lund, Sweden, daiva.daukantaite@psy.lu.se ORCID: https://orcid.org/0000-0002-1994-041X

**Keywords:** embodiment, Embodiment Scale-12 (ES-12), adolescence, body dissatisfaction, disordered eating, non-suicidal self-injury, anxiety, depression, incremental validity

## Abstract

**Background:**

Adolescence is characterized by large bodily changes and a heightened body-focus. It is also a sensitive period for the onset of various forms of psychopathology. Previous longitudinal studies have shown that body dissatisfaction is a predictor of disordered eating, non-suicidal self-injury (NSSI), and depression among adolescents. Body dissatisfaction, however, only represents one aspect of bodily self-experience. Another aspect is embodiment, defined as the anchoring of one’s identity in bodily self-experience. Research in this area, however, has been hampered by the lack of a psychometrically sound measure of embodiment that can be administered to adolescents. The purpose of the present study was to develop a brief measure of embodiment suitable for young adolescents.

**Methods:**

A 12-item Embodiment Scale (ES-12) was developed and underwent confirmatory factor analysis and tests of internal consistency, test-retest reliability, measure invariance, subscale inter-correlations, and construct validity. Incremental validity was analyzed to see if the ES-12 could predict disordered eating, non-suicidal self-injury, depression, and anxiety, above and beyond that of a measure of body dissatisfaction.

**Results:**

The ES-12 was found to exhibit robust psychometric properties, such as a distinct three-factor structure, strong internal consistency, and good test-retest reliability. It demonstrated good convergent and divergent validity, indicating that its three subscales—Harmonious Body, Disharmonious Body, and Body for Others—are significantly associated with a range of psychological health issues in adolescents. In addition, the ES-12 demonstrated consistent incremental validity by predicting disordered eating, NSSI, depression, and anxiety, beyond that of a measure of body dissatisfaction.

**Conclusions:**

The results suggest that ES-12 is a useful instrument in research on the experience of embodiment among adolescents.

## Background

Adolescence is a period characterized by large bodily changes and a heightened body-focus. This has been given particular attention in research on girls’ development. According to Piran’s (2016) developmental theory, for example, puberty is a period that poses many challenges to girls and may lead to a “crisis of embodiment” (p. 54). Adolescence is also a sensitive period for the onset of various forms of psychopathology (e.g., Guyer, 2020). Longitudinal studies show that *body dissatisfaction* predicts the development of disordered eating (e.g., Foster et al., 2024), non-suicidal self-injury (NSSI; Black et al., 2019), and depression (Blundell et al., 2024) during adolescence. But does body dissatisfaction capture all there is to the association between disturbances in bodily self-experience and psychopathology among adolescents? Several phenomenologically oriented psychiatrists (e.g., Fuchs, 2022; Fuchs & Schlimme, 2009; Stanghellini et al., 2019), have proposed that a focus on e*mbodiment* may add to the explanation of the role of bodily self-experience in the development of psychological problems.

Embodiment, as defined here, refers to the combined experience of *having* a body (that is publicly observable) and *being* this body (the body as felt “from within”) (Legrand, 2010; Lundh & Foster, 2024; Wehrle, 2020). Importantly, embodiment can be harmonious or disharmonious. For example, various forms of tension and conflict in bodily self-experience can appear in connection with the bodily transformations that take place during puberty (Leder, 1990; Osler, 2021). It is therefore a relevant research question if a focus on embodiment can help to advance our understanding of eating disorders and other forms of psychological health problems that develop during adolescence. This question is especially important in view of the increasing rates of mental ill-health that have been reported amongst young people (e.g., Haidt, 2024; Hay et al., 2023).

The study of how embodiment is related to the development of psychological health problems during adolescence requires valid measures of embodiment adapted to this age group. No measure of embodiment specifically for adolescents has so far been reported in the literature. The present study describes the development and validation of a brief embodiment scale suitable for adolescents and beyond.

### The Need for a Measure of Embodiment for Adolescents

Piran et al. (2020) have developed a 34-item Experienced Embodiment Scale (EES) for adults, which has shown good psychometric properties (Kling et al., 2020; Piran et al., 2020; Vankerckhoven et al., 2023). The length of EES and its rather complex language, however, speaks against its use among young adolescents. In addition, and essential to the purposes of the present study, we had concerns with some of the items in the EES. Because one of our purposes was to study the empirical associations between embodiment and disordered eating, we needed a measure of embodiment with items that are conceptually independent from disordered eating. From this perspective, one problem with the EES is that it contains items such as “My eating habits are a way for me to manage my emotions or how I have felt about myself” (item 7); “I engage in potentially harmful or painful behaviors (e.g., disordered eating, bingeing, purging, denying physical needs, skin-cutting, burning, drug use, excessive alcohol consumption” (item 15); and “I have an eating disorder” (item 16). These items ask about disordered eating (and even NSSI, as in item 15) rather than embodiment. The inclusion of such items in a measure of embodiment makes it less useful in research on the relationship between embodiment and disordered eating.^1^

Thus, the present study took its starting point in a felt need (a) for a shorter measure of embodiment to use in research on adolescents, with language more adapted to their age, which (b) does not overlap with items from measures of psychopathology (e.g., disordered eating, NSSI, depression). An additional theoretical consideration was that the items of this measure should refer to aspects of bodily self-experience that exemplify the concept of embodiment rather than other body-related constructs such as body image, body esteem, or body awareness. This required a theoretical clarification of the concept of embodiment, and of how it differs from these other body-related constructs. The following sections summarize our theoretical considerations in this regard.

### Embodiment as Differentiated from Body Image and Body Esteem

The most frequently used body-related constructs in research on disordered eating are *body image* and *body esteem* (Fairburn, 2008; Holmqvist Gattario & Frisén, 2019; Stice et al., 2021), with an emphasis on how the body is viewed and evaluated (e.g., Grogan, 2006). Relying on these constructs may, however, overlook even more profound changes in bodily experiences and self-awareness during adolescence, and therefore narrow down the possible conclusions that can be drawn (Fuchs, 2022). Anorexia nervosa, as argued by Fuchs (2022), is “a fundamental disturbance of embodied self-experience”, which involves a dis-identification with the body (or a conflictual relationship with the body), as illustrated by statements like “I feel caught in my body” and “My body is not me”.

Experienced embodiment, as we define it, is assumed to be fundamental to one’s feelings of self-identity. Among other things, this means that experienced embodiment involves an identification with one’s body, and that it can involve various kinds of conflicts in relation to the body and the extent to which the body is experienced as an object for others. The importance of the body for the experience of self-identity has been emphasized by many researchers. Erikson (1956, 1968), for example, described identity development as a gradual integration of different aspects of self, including that of bodily self-experience. As Erikson (1968) described it, an optimal experience of identity includes “a feeling of being at home in one’s body” (p. 165), which is a feeling that may be disturbed during periods when the body undergoes rapid change, such as for example in adolescence.

### Embodiment as Differentiated from Body Awareness

*Body awareness* is commonly divided into interoception and proprioception. Interoception is about sensations from inside the body, whereas proprioception is about movement, posture, and balance. Interoceptive deficits, defined as a deficient ability to sense the physiological conditions of the body and as measured by the Eating Disorder Inventory (EDI; Garner et al., 1983), have been found to be associated with eating disorders (Jenkinson et al., 2018).

According to Stanghellini et al. (2019), embodiment involves the holistic ability to *synthesize* interoceptions into a basic experience of self-consciousness and self-definition. They refer to this interoceptive capacity as *coenaesthesia*, defined as “the global experience in which all the single bodily sensations are synthesised” (p. 2) and advocate an “optical-coenaesthetic disproportion hypothesis”, according to which feeding and eating disorders are the result of an *imbalance* between coenaesthesia and the visually anchored body image. This hypothesis remains to be empirically tested and has been questioned by Fuchs (2022) for several reasons (p. 113*n*). Of most interest for the present purposes, however, is the conceptual point made by Stanghellini et al. (2019) that, although embodiment requires body awareness it also involves issues of *identity*.

Fuchs (2017) makes the additional point that “[if] the self is to persist in an unstable world, it must experience and have the capacity to accept its own body and feelings as its primordial selfhood” (p. 158). Or, in other words: healthy embodiment involves the ability to accept one’s bodily self-experience as basic to one’s identity.

A similar point regarding bodily self-experience and identity is made by Mehling et al. (2012), although from another theoretical perspective. According to these authors, body awareness may be either beneficial or maladaptive, depending on the attitude with which the body is attended to. For example, an attitude characterized by an exaggerated analytic focus on physical symptoms and a rumination about the meaning of physical symptoms is likely to be associated with dysfunctional forms of worry and anxiety. On the other hand, an attitude characterized by mindfulness, nonjudgmental acceptance, and the trusting of one’s body sensations goes together with “a sense of self grounded in experiencing physical sensations in the present moment, sometimes summarized as a sense of embodiment” (p. 1). The latter attitude is said to involve “an overall felt sense of an ‘embodied self’” (p. 3) – thus indicating a connection between embodiment, *mindful* body awareness, and identity.

Dahlberg (2019), in a partly similar manner, differentiates between two different kinds of body awareness in health and illness. According to her conceptualization, the more adaptive form involves a movement of *approaching* body sensations with a felt trust of the body. The less adaptive form, in contrast, involves a movement of *distancing* oneself from body sensations while mistrusting the body.

*Trusting* is also the name of one of the eight subscales in Mehling et al.’s (2018) Multidimensional Assessment of Interoceptive Awareness (MAIA-2). The other subscales are called Noticing, Non-Distracting, Not-Worrying, Attention Regulation, Emotional Awareness, Self-Regulation, and Body Listening. The Trusting subscale includes items that refer to trusting one’s sensations and feeling the body to be a “safe place” that one is “at home in”. In accordance with the reasoning in the present section a valid measure of embodiment may be expected to correlate with this scale, and also with the MAIA-2 subscales Self-Regulation (which contains items such as “When I bring awareness to my body I feel a sense of calm” and “I can use my breath to reduce tension”), Not-Distracting (e.g., not distracting oneself from sensations of discomfort) and Not-Worrying (e.g., being able to noticing an unpleasant body sensation without worrying about it). In contrast, the items of some of the other subscales, such as Body Listening and Noticing are more ambiguous, as they are about exploring and noticing body sensations – which may be done *either* with an analytic, ruminative, and non-trusting attitude or with a mindful, non-judgmental, trusting attitude. The items in these subscales may therefore be expected to be endorsed *both* by respondents who feel embodied in a harmonious way *and* by respondents who attend to their body sensations because of health-related worries and rumination about the meaning of various physical symptoms. This means that these scales may be expected to show at most a weak correlation with a measure of embodiment.

### The Present Study

To summarize, the first purpose of the present study was to develop a brief embodiment scale that can be used in research on adolescents, and to test its factor structure, reliability, and validity. The choice of items was guided by the theoretical considerations as described above. To summarize, this means that the items should:

- not overlap with items from measures of psychopathology (e.g., disordered eating, NSSI, depression),
- describe aspects of bodily self-experience that overlap as little as possible with other body-related constructs such as body image and body esteem; and
- capture aspects of harmonious versus non-harmonious ways of relating to one’s body, and aspects of identification versus dis-identification with one’s body.

It was expected that embodiment should correlate positively with well-being and negatively with measures of psychological health problems. It was also expected that embodiment would correlate positively with positive forms of body awareness, such as trusting the body, bodily self-regulation, non-worrying, and not-distracting, whereas it would at most correlate weakly with more ambiguous aspects of body awareness such as noticing and listening to the body.

A second purpose was to study the incremental validity (Haynes & Lench, 2003) of the embodiment scale, as compared with body satisfaction, in the prediction of disordered eating, NSSI, depression, and anxiety. As described above, previous research shows that body dissatisfaction predicts disordered eating, NSSI, and depression in adolescents. It was reasoned that, if the testing of incremental validity shows that the embodiment scale can explain the variance in disordered eating and other forms of psychopathology beyond that done by body dissatisfaction, this supports the value of the embodiment scale as a potentially useful instrument in research on the relation between embodiment and psychopathology.

A third purpose was to use the new embodiment scale in person-oriented analyses, to analyze individual profiles of embodiment and their association with psychological health problems. It was expected that factor analysis of the new embodiment scale would result in the construction of subscales, and that individual patterns of scores on these subscales could be used to identify subgroups of adolescents with different embodiment profiles. Because of the extensiveness of the study, however, it is divided into two papers with this third purpose being the subject of a separate paper (Lundh et al., 2025).

## Methods

**Figure A1:**
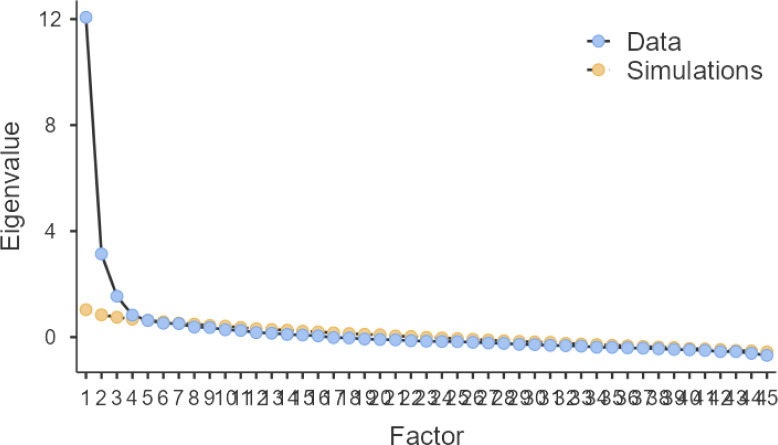
Scree Plot from Exploratory Factor Analysis Minimum Residual Extraction Method and Oblimin Rotation with Number of Factors Determined by Parallel Analysis

**Figure A2:**
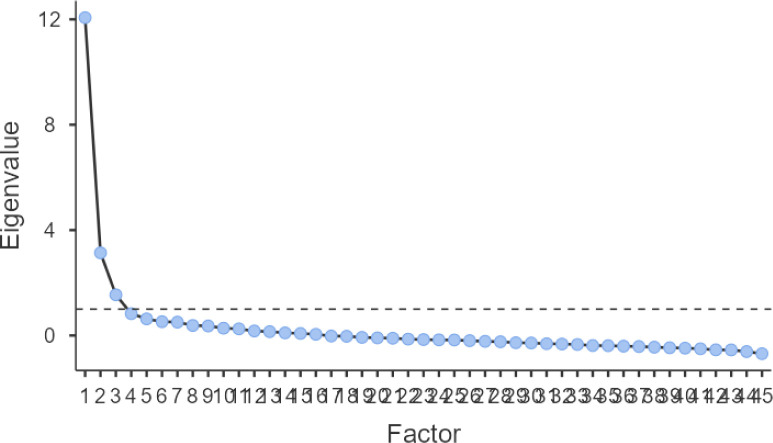
Scree Plot from Exploratory Factor Analysis Minimum Residual Extraction Method and Oblimin Rotation with Number of Factors Determined by Eigenvalue >1.

The development and testing of the new Embodiment Scale followed a three-stage process, which is described in more detail in the Appendix (see Tables A1-A3 and Figures A1-A2 for the results of exploratory factor analyses, and the final choice of items).

### Participants

#### Sample 1 – public junior high school – initial long version of the embodiment scale

Sample 1 comprised 323 adolescents (159 girls, 155 boys, 9 undisclosed or not identifying as either a girl or boy; 9 % with foreign background^2^) in 7th to 9th grade. Students’ ages ranged from 12 to 16, with a mean age of 14.17 years (*SD* = 0.96).

#### Sample 2 – public junior high school

Sample 2 comprised 238 adolescents (104 girls, 130 boys, 4 undisclosed or not identifying as either a girl or boy; 12.2% with foreign background) in grade 7 to 9. Students’ ages ranged from 13 to 16, with a mean age of 14.14 years (*SD* = 0.89). Test-retest data were obtained from 173 adolescents (74 girls, 96 boys, 3 undisclosed or not identifying as either a girl or boy; 10.4% with foreign background) from this sample, with a mean age of 14.22 years (*SD* = 0.87). The delay between the test and retest ranged from 28 to 35 days, yielding a response rate of 72.7%.

#### Sample 3 – public junior high school

Sample 3 comprised 292 adolescents (155 girls, 132 boys, 5 undisclosed or not identifying as either a girl or boy; 15.1% with foreign background) in grade 7 to 9. Students' ages ranged from 13 to 17 with a mean age of 14.32 years (*SD* = 0.89).

### Procedure

Students in public junior high school completed a digital survey using personal or school-provided laptops, tablets, or cellphones, with the survey link emailed to them by the researchers. Information about the project's aims and content, including details about confidentiality and the voluntary nature of participation, was sent to both students and their parents prior to data collection. This information emphasized that students were free to refrain from participating in the survey without providing reasons. Parents were informed that they could contact the project leader or class teacher to prohibit their child's participation in the survey. All participants provided digital consent to participate in the study before completing the survey.

Data collection took place during a designated lecture hour in the classroom. A clinically trained researcher and a research assistant administered the survey, while teachers were present to maintain order but did not participate in the administration process. Additionally, a clinically trained psychologist was available on-site, via phone or e-mail to address any iatrogenic effects or other problems and concerns that could arise during the survey or up to a week after data collection. Ethical approval was provided by the Swedish national ethics review board (registration numbers 2020-05885; 2021-06695-01; 2022-02093-02).

### Measures

#### Embodiment Scale (ES-12)

The 12-item Embodiment Scale (*ES-12*) contains 12 statements divided into three subscales; *Harmonious Body* (HB; e.g., “I feel at home in my body.”), *Disharmonious Body* (DB; e.g., “It happens that my body feels completely foreign to me.”), and *Body for Others* (BO; e.g., It is important to me what other people think about my appearance and physical characteristics.”). For a complete list of the items, see Table A5 in the Appendix. The respondents rate the items as how often they have the corresponding experiences, from 1 to 5 (where 1 means “never” and 5 means “very often”). Cronbach’s alpha values were as follows: HB = .85, DB = .77, BO = .79, and for the total scale .88.

#### Body esteem, self-esteem, and life satisfaction

*Body dissatisfaction* was assessed using the Body Esteem Scale for Adolescents and Adults – Appearance subscale (BESAA; Mendelson et al., 2001), which contains 10 statements (e.g., “I look as good as I’d like”). *Self-esteem* was assessed using Rosenberg’s Self-Esteem Scale (RSES; Rosenberg, 1989), which consists of 10 items (e.g., “On the whole, I am satisfied with myself”). *Life satisfaction* was assessed using the Students’ Life Satisfaction Scale (SLSS; Huebner, 1991), which consists of six items (e.g., “My life is going well”).

#### Body awareness

*Interoceptive awareness* was assessed using the Multidimensional Assessment of Interoceptive Awareness 2 (MAIA-2; Mehling et al., 2018). This scale comprises 32 items that assess eight facets of interoceptive body awareness: Noticing, Non-distracting, Not-worrying, Attention regulation, Emotional awareness, Self-regulation, Body listening, and Trusting.

#### Measures of psychological health problems

*Psychological difficulties* were assessed using four subscales from the Strengths and Difficulties Questionnaire – self-report version (SDQ-s; Goodman, 1997; Lundh et al., 2008): Hyperactivity/inattention (e.g., “I am easily distracted, I find it difficult to concentrate”), Emotional symptoms (e.g., “I worry a lot”), Conduct problems (e.g., “I get very angry and often lose my temper”) and Peer problems (e.g., “Other children or young people pick on me or bully me”).

*Depression* and *anxiety* were assessed using a 25-item version of Revised Children’s Anxiety and Depression Scale (RCADS-25; Chorpita et al., 2000; Ebesutani et al., 2012), which contains two subscales to measure anxiety and depression. The Anxiety subscale consists of 15 items (e.g., “I worry when I think I have done poorly at something”) and the Depression subscale consists of 10 items (e.g., “Nothing is much fun anymore”).

*Disordered eating* (DE) was assessed by two different measures: (1) The Risk Behaviour related to Eating Disorders (RiBED-8; Waaddegaard et al., 2003; Viborg et al., 2012), which contains eight items on behaviours and attitudes related to food consumption (e.g., “I vomit to rid myself of food I have eaten”). The respondents are asked to rate how often they engage in these on a scale from 1 (“never”) to 4 (“very often”), and a total score is calculated by adding the scores on all items to a total score (range 8-32). (2) The SCOFF questionnaire (Hansson et al., 2016; Morgan et al., 1999), which contains five questions concerning eating habits and attitudes toward weight and body shape, that are answered in a yes/no format (e.g., “Do you believe yourself to be fat when others say you are thin?”). A total score is computed as the number of questions that were given a positive answer. SCOFF and RiBED-8 showed a correlation of r = .76 in the present study.

*Non-suicidal self-injury* was assessed with a 9-item version of the Deliberate Self-Harm Inventory (DSHI-9r; Gratz, 2001; Lundh et al., 2011), where the respondents are asked to indicate how often they have deliberately injured themselves (e.g., by cutting, carving, or severely scratching themselves, or preventing wounds from healing) in the past 6 months. This is done on a scale from 0 (never) to 6 (more than five times), and a total score (range 0−54) is computed by summing all items.

### Statistical Analyses

Confirmatory factor analysis (CFA) was employed, utilizing robust maximum-likelihood estimation with data from Sample 3, to evaluate the 3-factor structure suggested by the exploratory factor analysis of the ES-12 from Sample 2. Model fit was assessed using the Root Mean Square Error of Approximation (RMSEA) with 90% confidence intervals, the Comparative Fit Index (CFI), the Tucker–Lewis Index (TLI), and the Standardized Root Mean Residual (SRMR). Acceptable model fit was defined as RMSEA ≤ .08, CFI and TLI ≤ .90, and SRMR ≤ .08, while good model fit was defined as RMSEA ≤ .05, CFI and TLI ≥ .95, and SRMR ≤ .05 (Hooper et al., 2008; Hu & Bentler, 1999). To assess measurement invariance across genders, we compared more restrictive models to a less constrained model, focusing on changes in CFI (ΔCFI) and RMSEA (ΔRMSEA). Consistent with previous research (Chen, 2007; Sass, 2016), we considered ΔCFI ≤ .010 and ΔRMSEA ≤ .015 as indicators of the invariance assumption being met for concluding metric and scalar invariance.

Internal consistency was estimated using Cronbach’s *α* and McDonald’s *ω* levels, with a criterion value of >.70 for acceptable consistency (DeVellis, 2003). Test-retest reliability coefficients were computed for all subscales using data from Sample 2. Convergent and discriminant validity were examined using Pearson correlations, following Cohen’s (1988) guidelines for interpretation, where correlations *r*

≥ .50 were considered large, *r* = .30 – .50 as medium, and *r* < .30 as small.

To assess the incremental validity of the ES-12, hierarchical multiple regressions were conducted to predict the degree of disordered eating and other psychopathology-related measures, including NSSI, symptoms of anxiety, and depression, after controlling for the degree of body dissatisfaction. Preliminary analyses ensured no violations of normality, linearity, multicollinearity, or homoscedasticity assumptions.

The statistical analyses were conducted using jamovi (Version 2.3), Mplus Version 8 (Muthén & Muthén, 1998-2017) and SPSS Statistics 28.

## Results

### Factor Structure

**Figure 1:**
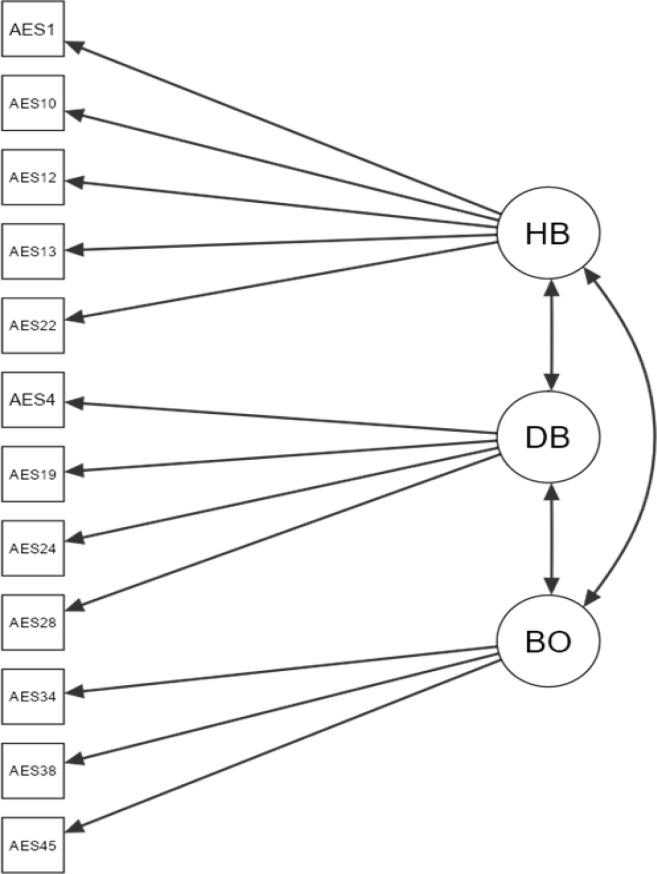
Three Factor ES-12 Model with 12 Indicator Items. *Note.* HB = *Harmonious Body*; DB = *Disharmonious Body*; BO = *Body for Others*. See Table A5 in the Appendix for the contents of the items.

Confirmatory factor analysis of the ES-12 was conducted with data from Sample 3. Four models were evaluated and compared based on fit indices (see Table 1). The single-factor model demonstrated inadequate goodness of fit (CFI = .76; TLI = .71; RMSEA = .15; SRMR = .10), as did a two-factor model with one factor assessing *Harmonious Body* and *Disharmonious Body* and the other factor assessing *Body for Others* (CFI = .89; TLI = .86; RMSEA = .11; SRMR = .07). In contrast, the three-factor model exhibited good fit across all indices (CFI = .96; TLI = .95; RMSEA = .06; SRMR = .06). However, the bifactor model with three factors and a general factor demonstrated superior fit (CFI = .98; TLI = .97; RMSEA = .05; SRMR = .04) and marginally outperformed the three-factor model (ΔCFI = .02, ΔTLI = .02, ΔRMSEA = .01, ΔAIC = −12.2). The loadings and covariances of the bifactor model are depicted in Figure 1.

**Table 1:** Results of Confirmatory Factor Analysis of the 12-item Embodiment Scale (ES-12). Model Comparison.

Model	*χ2*	df	CFI	TLI	RMSEA (90%)	SRMR	AIC
1-factor model	414.7	54	.76	.71	.15 (.14-.17)	.10	9628.6
2-factor model	220.9	53	.89	.86	.11 (.09-.12)	.07	9436.7
3-factor model	105.5	51	.96	.95	.06 (.04-.08)	.06	9325.3
Bifactor model	75.3	42	.98	.97	.05 (.03-.07)	.04	9313.1

*Note.* N = 290; CFI = comparative fit index; TLI = Tucker Lewis index; RMSEA = root mean square error of approximation; SRMR = standardized root mean residual; AIC = Akaike's information criterion. The 2-factor model included one factor assessing *Harmonious Body* and *Disharmonious Body*, and the other factor assessing *Body for Others*.

### Internal Consistency

Table 2 presents the internal consistency values (Cronbach’s alpha and McDonald's omega) of *ES-12* for all samples. Across all individual samples and when combined, alpha values exceeded .70, indicating good internal consistency.

**Table 2:** Cronbach’s Alpha/McDonald’s Omega Values for ES-12 Subscales and the Total ES-12 scale Across Samples.

Subscale/scale	Sample			Combined
	1	2	3	
Harmonious body	.80/.82	.85/.86	.84/.85	.84/.86
Disharmonious body	.73/.74	.78/.77	.77/.77	.73/.74
Body for others	.82/.82	.81/.82	.75/.80	.78/.80
ES-12, total	.88/.87	.89/.89	.87/.87	.87/.87

### Test-Retest Reliability

All test-retest correlations were medium to large (*n* = 173): *r* [95% CI] *Harmonious Body* = .83 [.77, .87], *Disharmonious Body* = .69 [.60, .76], and *Body for Others* = .72 [.64, .78].

### Measurement Invariance

Table A5 in the Appendix summarizes the results for method invariance across genders. We found evidence of scalar invariance between girls and boys across all fit indices of interest indicating that mean values are directly comparable across the genders.

### Subscale Intercorrelations

As to the intercorrelations among the ES-12 subscales, *Harmonious Body* showed negative correlations with both *Disharmonious Body* (*r* = -.60, p < .001) and *Body for Others* (*r* = -.45, p < .001), whereas the latter two showed a moderate positive correlation (*r* = .41, p < .001).

### Construct Validity

Table 3 shows how the three ES-12 subscales and the total score correlated with the other variables. As to convergent validity, *Harmonious Body* correlated positively with body satisfaction, life satisfaction and self-esteem, and negatively with all psychopathology-related measures. *Disharmonious Body* and *Body for Others*, in contrast, correlated negatively with body satisfaction, life satisfaction and self-esteem, and positively with all psychopathology-related measures. As to the size of the correlations, the *ES-12* subscales showed medium to strong correlations with all measures of disordered eating, NSSI, anxiety, depression, and emotional problems, except for the weak correlation between NSSI and Body for Others (*r* = .22). The *ES-12* subscales showed only weak correlations with hyperactivity-inattention, conduct problems, and peer problems.

**Table 3:** Correlations of ES-12 and its Subscales with the Other Variables (N = 471-516).§

	*HB*	*DB*	*BO*	*ES-12 total*
**BESAA**	.80	-.61	-.56	.83
**RSES**	.74	-.65	-.41	.74
**Life Satisfaction**	.66	-.58	-.38	.68
**MAIA-2**				
Noticing	.07	.12	.14	.11
Non-distracting	.17	-.23	-.21	.23
Not-worrying	.21	-.27	-.30	.31
Attention regulation	.06	-.01	.12	.01
Emotional awareness	-.02	.13	.18	-.10
Self-regulation	.33	-.24	-.17	.31
Body listening	.24	-.09	-.09	.19
Trusting	.78^a^	-.62	-.38	.73
**SDQ-s**				
Hyperactivity/inattention	-.24	.23	.17	-.25
Conduct problems	-.21	.23	.14	-.22
Emotional symptoms	-.57	.62	.41	-.65
Peer problems	-.21	.29	.06	-.22
**RCADS-25**				
Anxiety	-.55	.60	.51	-.67
Depression	-.63	.59	.36	-.65
**RIBED-8**	-.66	.53	.49	-.70
**SCOFF**	-.55	.42	.41	-.56
**DSHI-9r**	-.45	.42	.22	-.43

Correlations *r* > .14 are significant at *p* < .001

a *r* = .20 if partly overlapping item is excluded

*Note.* HB = Harmonious Body; DB = Disharmonious Body; BO = Body for Others; BESAA = Body Esteem Scale for Adolescents and Adults – Appearance subscale; RSES = Rosenberg’s Self-Esteem Scale; MAIA-2 = Multi-dimensional Assessment of Interoceptive Awareness 2; SDQ-s = Strength and Difficulties Questionnaire – self-report version; RCADS-25 = Revised Child Anxiety and Depression Scale shortened version; RIBED-8 = Risk Behaviour related to Eating Disorders; DSHI-9r = Deliberate Self-Harm Inventory – 9 item version.

As to the measures of body awareness, the *ES-12* subscales showed consistently positive and statistically significant correlations with four of the eight MAIA-2 subscales: whereas they showed mostly negligible correlations with the others. As seen in Table 3, the largest correlations were found with the subscale Trusting. It should be noted, however, that the large correlation (*r* = .78) between ES-12 *Harmonious Body* and MAIA-2 Trusting was due to a partial overlap between items in these two subscales: “I feel at home in my body” in *Harmonious Body* and “I am at home in body” in the MAIA-2 *Trusting* subscale. When these items were excluded, so that ES-12 *Harmonious Body* was reduced to a 4-item subscale and MAIA-2 Trusting was reduced to a two-item subscale, the correlation was reduced from *r* = .78 to *r* = .20 but was still statistically significant (*p* < .001).

### Incremental Validity

To test the incremental validity of the ES-12 subscales, we carried out a series of hierarchical multiple regressions to assess their ability to predict degree of disordered eating and other psychopathology-related measures after controlling for degree of body dissatisfaction. The results are seen in Table 4.

**Table 4:** Results of Multiple Regressions Predicting Psychological Health Problems from Body Dissatisfaction and Embodiment.

	**Disordered Eating (RiBED-8)**		
	***B*(*SE*)**	***β***	***R*2**

**Step 1**			.43***
BESAA	-0.42 (0.02)	-.66***	
**Step 2**			.51***
BESAA	-0.15 (0.04)	-.23***	
ES-12 HB	-1.77 (0.29)	-.34***	
ES-12 DB	0.76 (0.29)	.11**	
ES-12 BO	0.79 (0.18)	.17***	

	**Disordered Eating (SCOFF)**		
	*B* (*SE*)	*β*	*R*2

**Step 1**			.26***
BESAA	-0.08 (0.01)	-.51***	
**Step 2**			.33***
BESAA	-0.01 (0.01)	-.09	
ES-12 HB	-0.44 (0.08)	-.37***	
ES-12 DB	0.07 (0.08)	.05
ES-12 BO	0.19 (0.05)	.17***	

	**Non-suicidal self-injury (DSHI-9r)**		
	*B* (*SE*)	*β*	*R*2

**Step 1**			.18***
BESAA	-0.48 (0.05)	-.43***	
**Step 2**			.25***
BESAA	-0.15 (0.08)	-.14	
ES-12 HB	-2.05 (0.62)	-.22***	
ES-12 DB	2.81 (0.61)	.23***	
ES-12 BO	-0.44 (0.39)	-.05	

	**Depression (RCADS-25)**		
	*B* (*SE*)	*β*	*R*2

**Step 1**			.39***
BESAA	-0.46 (0.03)	-.62***	
**Step 2**			.47***
BESAA	-0.19 (0.04)	-.26***	
ES-12 HB	-1.54 (0.47)	-.26***	
ES-12 DB	2.03 (0.33)	.26***	
ES-12 BO	-0.06 (0.21)	-.01	

	**Anxiety (RCADS-25)**		
	*B* (*SE*)	*β*	*R*2

**Step 1**			.33***
BESAA	-0.57 (0.04)	-.58***
**Step 2**			.46***
BESAA	-0.13 (0.06)	-.13*	
ES-12 HB	-1.27 (0.47)	-.14*	
ES-12 DB	3.51 (0.46)	.33***	
ES-12 BO	1.68 (0.29)	.23***	

* *p* <

** .05; **p < .01;

*** *p* < .001

*Note.* BESAA = Body Esteem Scale for Adolescents and Adults – Appearance subscale; ES-12 = Embodiment Scale-12; HB = Harmonious Body; DB = Disharmonious Body; BO = Body for Others; RiBED-8 = Risk Behaviour related to Eating Disorders; DSHI-9r – Deliberate Self-Harm Inventory – 9 item version; RCADS-25 = Revised Child Anxiety and Depression Scale - shortened version.

#### Disordered eating

Disordered rating was measured both by RiBED-8 and SCOFF, which correlated *r* = .76 in the present study. The regression with RiBED-8 as the dependent variable is shown in Table 5. After entering the *ES-12* subscales at Step 2, the total variance explained by the model was 50.9%, *F*(4, 483) = 125.29, *p* < .001. The *ES-12* subscales explained an additional 8% of the variance in RiBED-8 after controlling for degree of body dissatisfaction, *ΔR^2^* = .08, *ΔF* (3, 483) = 25.65, *p* < .001. In the final model all four variables were statistically significant, with the ES-12 *Harmonious Body* subscale recording the highest beta value, *β* = -.34, *p* < .001 (see Table 4).

The regression with SCOFF as the dependent variable is shown in Table 4. After entering the *ES-12* subscales at Step 2, the total variance explained by the model was 33.2%, *F*(4, 486) = 60.44, *p* < .001. The *ES-12* subscales explained an additional 8% of the variance in SCOFF after controlling for degree of body dissatisfaction, *ΔR^2^*= .08, *ΔF* (3, 486) = 18.17, *p* < .001. In the final model only two of the variables were statistically significant: the subscales *Harmonious Body* (*β* = -.37, *p* < .001) and *Body for Others* (*β* =.17, *p* < .001).

#### Non-suicidal self-injury (NSSI)

The regression with NSSI as the dependent variable is shown in Table 4. After entering the *ES-12* subscales at Step 2, the total variance explained by the model was 24.6%, *F*(4, 493) = 40.14, *p* < .001. The *ES-12* subscales explained an additional 7% of the variance in NSSI after controlling for degree of body dissatisfaction, *ΔR2* = .07, *ΔF*(3, 493) = 14.13, *p* < .001. In the final model only two of the variables were statistically significant: the *ES-12* subscales *Harmonious Body* (*β* = -.22, *p* < .001) and *Disharmonious Body* (*β* = .23, *p* < .001).

#### Depression

The regression with the RCADS Depression subscale as the dependent variable is shown in Table 4. After entering the *ES-12* subscales at Step 2, the total variance explained by the model was 47.2%, *F*(4, 493) = 109.31, *p* < .001. The *ES-12* subscales explained an additional 8% of the variance in depression after controlling for degree of body dissatisfaction, *ΔR^2^* = .08, *ΔF* (3, 493) = 25.53, *p* < .001. In the final model three of the variables were statistically significant, with *ES-12 Harmonious, ES-12 Disharmonious Body,* and body satisfaction recording the highest beta values.

### Anxiety

The regression with the RCADS Anxiety subscale as the dependent variable is shown in Table 4. After entering the *ES-12* subscales at Step 2, the total variance explained by the model was 46.3%, *F*(4, 487) = 105.07, *p* < .001. The *ES-12* subscales explained an additional 13% of the variance in anxiety after controlling for degree of body dissatisfaction, *ΔR^2^* = .13, *ΔF* (3, 487) = 39.86, *p* < .001. In the final model, the variables with the highest beta values were the two ES-12 subscales *Disharmonious Body* (*β* = .33, *p* < .001) and *Body for Others* (*β* = .23, *p* < .001).

## Discussion

The present findings suggest that the newly developed 12-item Embodiment Scale for Adolescents (*ES-12*) exhibits robust psychometric properties, such as a distinct three-factor structure, strong internal consistency, and good test-retest reliability. Additionally, the *ES-12* demonstrates good convergent and divergent validity, indicating that its three subscales—Harmonious Body, Disharmonious Body, and Body for Others—are significantly associated with a range of psychological health issues. Furthermore, the *ES-12* demonstrates consistent incremental validity by predicting measures of disordered eating, NSSI, depression, and anxiety, beyond what is accounted for by the measure of body dissatisfaction. This suggests that the *ES-12* captures facets of bodily self-experience relevant to psychopathology that measures of body dissatisfaction may overlook. These findings align with theoretical frameworks that emphasize the importance of the concept of embodiment for the understanding of various forms of psychopathology (Fuchs, 2022; Fuchs & Schlimme, 2009).

### Construct Validity

The *ES-12* subscale Harmonious Body was found to correlate negatively with Disharmonious Body and Body for Others, whereas the latter two were positively correlated. This is in accordance with theoretical assumptions that a focus on how one’s body is viewed by others is inversely related to harmonious forms of embodiment. The literature contains various possible explanations for this inverse relationship. One possibility is that the internalization of the external gaze is disruptive to the experience of embodiment (Piran & Teall, 2012); another suggestion is that the individual seeks the other’s gaze as a compensation for poor experiences of embodiment (Stanghellini et al., 2019).

It might be questioned whether Body for Others really belongs to the construct of embodiment or should rather be seen as a separate construct that is empirically associated with Embodiment. It is possible, however, to find arguments for why it should be seen as part of the embodiment construct. The three items in this subscale (see Table A3) all refer to how others think about one’s appearance and physical characteristics, and the importance assigned to this. The latter means that these items are not mere expressions of one’s body image, body esteem, or degree of body dissatisfaction, but involve a more complex relationship to one’s body that may involve issues of self-identity. The body-for-others is also an important focus in the writings of embodiment researchers. Fuchs (2022), for example, describes “the basic polarity of embodiment” as involving, among other things, a polarity “between being-for-oneself and being-for-others” (p. 110). According to Stanghellini et al. (2019), a common experience among people with embodiment disorders is that “[t]he way they feel looked at by the others is the principal mode to feel themselves and define their identity” (p. 6). Finally, the confirmatory factor analysis showed that the bifactor model with three factors and a general factor demonstrated superior fit and marginally outperformed the threefactor model – again, consistent with the conceptualization of Body for Others as an integrative part of the embodiment construct.

### Convergent and Discriminant Validity

The convergent and discriminant validity of the *ES-12* subscales was studied in relation to measures of well-being, psychopathology, and various aspects of body awareness. In accordance with expectations, Harmonious Body correlated positively with measures of well-being and negatively with measures of psychopathology, whereas Disharmonious Body and Body for Others correlated negatively with measures of well-being and positively with measures of psychopathology. Most of these correlations were medium to large. One exception, however, was the low correlation (*r* = .22) between NSSI and Body for Others; this suggests that this dimension may be of less importance for individuals who engage in NSSI. Other exceptions were the correlations between the *ES-12* subscales and measures of Hyperactivity/Inattention, Conduct Problems, and Peer Problems; although these correlations all went in the expected direction, they were low (*r* < .30), thereby suggesting that experienced embodiment is less associated with these problems than with disordered eating, NSSI, depression, and anxiety.

As to the measures of Body Awareness (Mehling et al., 2018), most correlations with embodiment were low. One major exception was the MAIA-2 Trusting scale, which showed large correlations with Harmonious Body (*r* =.78) and Disharmonious Body (*r* = -.62) and a moderate correlation with Body for Others (*r* = -.38). The large correlation between Trusting and Harmonious Body, however, should not be taken at face value, as it was due to a partial overlap of content between items of these two subscales: “I feel at home in my body” in the ES-12 subscale Harmonious Body and “I am at home in body” in the MAIA-2 subscale Trusting. When these two items were excluded, the correlation was strongly reduced.

It should be noted, though, that the strong negative correlation between the *ES-12* subscale Disharmonious Body and the MAIA-2 subscale Trusting could not be explained in terms of item overlap. The need for more research on body trust, and the development of more elaborate measures of body trust, has recently been argued for by Grunewald et al. (2024). Here it is of interest that their newly developed Body Trust Scale does not contain any item on being/feeling at home in one’s body. Maybe this kind of item fits better as part of an Embodiment scale than as part of a scale measuring Body Trust, as it can be said to describe aspects of one’s identification with the body – that is, feeling at home in one’s body, implying a kind of identification with the body (Erikson, 1968).

### Incremental Validity

One of the most interesting results of the present study is the incremental validity of the *ES-12* subscales, as compared with the measure of body dissatisfaction, in relation to measures of psychopathology. Body dissatisfaction is known to be an important predictor, both cross-sectionally and longitudinally, of disordered eating (Foster et al., 2024; Stice et al., 2011), NSSI (Black et al., 2019), and depression (Blundell et al., 2024). Although the present study only involves an analysis of cross-sectional associations, it is nevertheless remarkable that the *ES-12* showed consistent incremental validity by predicting all measures of disordered eating, NSSI, depression, and anxiety, above and beyond the variance accounted for by the measure of body dissatisfaction. This clearly suggests that the *ES-12* captures other aspects of bodily self-experience of relevance for psychopathology than those captured by measures of body dissatisfaction. This is a promising finding that should be followed up in future research.

Interestingly, the *ES-12* subscales showed the highest beta values in the regression models for the prediction not only of disordered eating but also of NSSI, depression, and anxiety; only in predicting depression did body dissatisfaction reach a similar beta value. In the prediction of disordered eating, as measured by RiBED-8, Harmonious Body showed the highest beta value, although body dissatisfaction and the two other *ES-12* scales also contributed significantly to the final model. And with SCOFF as the measure of disordered eating only Harmonious Body and Body for Others contributed significantly to the final model. As to NSSI and depression, only Harmonious Body and Disharmonious Body contributed significantly to the final model. And in the prediction of anxiety, Disharmonious Body and Body for Others made the largest contribution.

The details of these results should be taken with caution; they need to be replicated before any conclusions can be drawn. The larger picture obtained from these results, however, clearly suggests that the *ES-12* captures other aspects of bodily experiences (aspects of experienced embodiment) that may possibly be even more important than body dissatisfaction for the development of disordered eating and other aspects of psychopathology.

### Limitations and Future Research

A main limitation of the present study is that we used only non-clinical samples. The present findings need to be replicated in clinical samples before any conclusions can be drawn about the clinical usefulness of the *ES-12*. Another limitation that has already been mentioned is that the present data are merely cross-sectional; future research should address questions about the incremental validity of *ES-12*, as well as the meaning of various embodiment profiles, also in the prospective studies using longitudinal samples. It is important to remember that the present results do not allow for any conclusions about causality or risk factors in a developmental perspective. Stronger conclusions must wait until longitudinal data are available for analysis.

A caution is also that our study may be susceptible to an increased Type 1 error due to the numerous analyses conducted. Nevertheless, it is pertinent to note that most of our correlational results remain significant at *p* < .001, which helps mitigate the risk associated with multiple comparisons. We have deliberately highlighted only those correlational results meeting this stringent threshold, further reducing the risk associated with multiple comparisons.

A final limitation concerns the items of the *ES-12*. Although, as described in the introduction, we took care to differentiate the embodiment construct as clearly as possible from other body-related constructs such as body image, body esteem, and body awareness, the question remains whether the subscale Body for Others really captures aspects of embodiment or should rather be seen as a separate construct. The question also needs to be raised whether there are other important aspects of embodiment that we have failed to capture in *ES-12*. For example, we have no items about moments of quiet bodily harmony when the body is not in direct focus, and we have no items referring to personal bodily expressiveness (e.g., the ability to move spontaneously and let go of self-focus when entering rhythms in dancing and music). Although the present scale was developed specifically for adolescents, the question also remains if it can be meaningfully used for adults, or if this would require some modification. All this points to a continued need to refine and improve our ways of measuring embodiment.

## Data Availability

The data that support the findings of this study are available on request from the corresponding author. The data are not publicly available due to privacy or ethical restrictions.

## Footnotes

1 The inclusion of these items in Piran et al.’s (2020) EES reflects an over-inclusive definition of “embodiment”, as exemplified in passages such as the following: “the construct of embodiment encompasses a broad range of experiences, including behavioral and psychological phenomena commonly conceptualized and studied separately, such as disordered eating, self-injury, substance use, or the practice of sexuality with no desire or without the use of protection” (Piran et al., 2020, p. 117). In contrast, we define embodiment in *phenomenological* terms, which makes it possible to study how experienced embodiment is *empirically associated* with various kinds of *behavioural* phenomena such as disordered eating, nonsuicidal self-injury, etc.

2 defined as the child either being born abroad with at least one parent born abroad, or being born in Sweden with both parents being born abroad.

## Action editor

Lars R. Bergman served as action editor for this article.

## Open access

This article is distributed under the terms of the Creative Commons Attribution 4.0 International License (http://creativecommons.org/licenses/by/4.0/), which permits unrestricted use, distribution, and reproduction in any medium, provided you give appropriate credit to the original author(s) and the source, provide a link to the Creative Commons license, and indicate if changes were made.

## Appendix

### The development of items for the new embodiment scale

The development and testing of the new embodiment scale followed a three-stage process. First, a comprehensive theoretical review of the concept of embodiment was conducted (see Lundh & Foster, 2024). Following this, the authors, in collaboration with other members of the research team, generated potential items for inclusion in the embodiment scale. This process was informed by an extensive review of existing surveys, and feedback on proposed items was also collected directly from adolescent participants to ensure relevance and clarity. As to the response format, we wanted the respondents to assess how often they had various experiences, rather than whether they agreed with various statements (i.e., beliefs). We therefore chose a response format where the respondents rated the items as how often they had the corresponding experiences, from 1 to 5 (where 1 meant “never” and 5 meant “very often”).

As a result of this first developmental phase, 45 items from the original pool of items were selected for a first version of the embodiment scale to be tested by means of exploratory factor analysis (Sample 1). This led to the identification of three factors, and the number of items was reduced to 13. In the second stage, this 13-item version was administered to a new sample (Sample 2) and subjected to exploratory factor analysis and preliminary psychometric evaluation, including test-retest reliability. These analyses led to the exclusion of one of the 13 items, thereby reducing the final version to 12 items. This 12-item version, named Embodiment Scale-12 (ES-12), was then subjected to confirmatory factor analysis in Sample 3. In the third stage, the construct validity of ES-12 was studied in the combined Samples 2 and 3.

### Initial Exploratory Factor Analysis of the 45-item Version

An exploratory factor analysis, employing oblimin rotation, was conducted on the data from Sample 1. Initially, three factors were identified based on eigenvalues exceeding 1, and four factors were indicated using parallel analysis. Nevertheless, in line with the scree plots, three main factors consistently emerged in both analyses, tentatively named *Harmonious Body, Disharmonious Body*, and *Body for Others*. The authors then independently chose five items from factor 1 and 2, along with three items from factor 3, considering the items’ theoretical importance and strong loading. Comprehensive preliminary findings from the exploratory factor analyses can be found in Tables A1 and A2, accompanied by the scree plots illustrating the two versions of factor determination methods in Figures A1 and A2.

**Table A1:** Factor Loadings in the Exploratory Factor Analysis: Minimum Residual Extraction Method and Oblimin Rotation with Number of Factors Determined by Parallel Analysis.

	Factor				Uniqueness
Item	1	2	3	4	
ES10	**0.75**	-0.10	-0.06	-0.01	0.33
ES13	**0.73**	-0.03	-0.11	-0.01	0.37
ES20	**0.73**	0.08	-0.15	0.00	0.40
ES2	**0.72**	-0.04	-0.04	-0.08	0.48
ES1	**0.66**	-0.05	-0.07	-0.01	0.51
ES15	**0.64**	-0.19	0.10	0.13	0.45
ES26	**0.59**	-0.01	-0.13	0.04	0.54
ES3	**0.58**	0.02	-0.06	0.13	0.55
ES18	**0.55**	-0.08	-0.02	0.22	0.49
ES39	**0.50**	0.09	0.06	0.25	0.62
ES14	**-0.45**	0.28	0.22	-0.01	0.44
ES41	**0.44**	0.12	0.19	0.18	0.75
ES12	**0.43**	-0.22	0.10	0.12	0.69
ES22	**0.41**	-0.15	0.03	0.10	0.73
ES43	0.33	0.16	0.31	-0.01	0.84
ES34	-0.03	**0.80**	0.03	-0.00	0.32
ES33	-0.09	**0.75**	-0.07	0.04	0.45
ES45	0.01	**0.74**	0.03	-0.07	0.43
ES38	-0.03	**0.67**	-0.01	0.12	0.54
ES36	-0.12	**0.52**	0.15	-0.24	0.43
ES37	0.05	**0.49**	-0.02	0.28	0.71
ES35	-0.05	**0.46**	0.26	-0.10	0.54
ES21	0.32	**0.40**	-0.09	0.12	0.79
ES28	-0.23	0.04	**0.60**	-0.02	0.42
ES27	-0.34	0.11	**0.54**	0.09	0.40
ES6	-0.07	-0.14	**0.45**	0.14	0.85
ES24	0.01	0.22	**0.43**	-0.25	0.54
ES19	-0.21	0.02	**0.43**	0.00	0.69
ES8	-0.15	0.09	**0.43**	-0.19	0.57
ES7	-0.11	-0.06	**0.42**	-0.13	0.74
ES4	-0.10	-0.03	**0.42**	-0.08	0.77
ES42	0.38	0.18	**0.40**	0.02	0.76
ES29	0.01	0.18	**0.40**	-0.07	0.72
ES31	-0.03	0.16	0.39	-0.28	0.59
ES30	-0.27	0.18	0.38	-0.01	0.56
ES44	-0.07	0.07	0.37	-0.26	0.67
ES40	0.05	0.14	0.35	-0.08	0.80
ES5	-0.04	-0.07	0.34	-0.00	0.89
ES25	-0.03	-0.22	0.33	-0.13	0.87
ES32	-0.16	0.31	0.32	-0.16	0.51
ES23	-0.18	0.13	0.25	0.06	0.82
ES16	0.15	0.04	0.04	**0.76**	0.33
ES17	0.24	0.03	0.20	**-0.70**	0.51
ES11	0.32	0.03	0.05	**0.62**	0.38
ES9	0.05	0.10	0.16	**0.29**	0.89

*Note*. Items highlighted in yellow, violet, and green have been selected for a concise version of the ES subscales: *Harmonious Body, Body-for-Others*, and *Disharmonious Body*, respectively.

**Table A2:** Factor Loadings in the Exploratory Factor Analysis: Minimum Residual Extraction Method and Oblimin Rotation with Number of Factors Determined by Eigenvalue >1.

	Factor			Uniqueness
Item	1	3	3	
ES10	**0.77**	-0.01	-0.13	0.34
ES13	**0.74**	-0.05	-0.07	0.39
ES15	**0.74**	0.04	-0.10	0.45
ES2	**0.72**	0.07	-0.11	0.50
ES20	**0.71**	-0.07	0.02	0.43
ES1	**0.67**	-0.01	-0.08	0.52
ES18	**0.64**	-0.13	0.03	0.49
ES3	**0.63**	-0.09	0.05	0.55
ES26	**0.60**	-0.10	-0.03	0.55
ES39	**0.59**	-0.04	0.20	0.63
ES41	**0.53**	0.14	0.21	0.76
ES12	**0.52**	0.02	-0.12	0.70
ES11	**0.51**	-0.30	0.33	0.49
ES22	**0.47**	-0.03	-0.09	0.73
ES14	**-0.47**	0.23	0.28	0.44
ES43	0.38	0.38	0.16	0.83
ES24	-0.02	**0.62**	0.11	0.54
ES17	0.05	**0.61**	-0.29	0.67
ES31	-0.07	**0.59**	0.04	0.59
ES28	-0.15	**0.58**	0.12	0.47
ES8	-0.15	**0.54**	0.04	0.57
ES44	-0.09	**0.53**	-0.02	0.67
ES29	0.03	**0.47**	0.17	0.72
ES7	-0.08	**0.47**	-0.06	0.75
ES32	-0.20	**0.46**	0.23	0.51
ES27	-0.25	**0.46**	0.23	0.46
ES42	0.44	**0.45**	0.21	0.76
ES4	-0.05	**0.44**	-0.00	0.78
ES40	0.07	**0.43**	0.12	0.80
ES35	-0.09	**0.40**	0.37	0.54
ES19	-0.15	**0.40**	0.08	0.72
ES36	-0.23	0.39	0.34	0.48
ES30	-0.24	0.39	0.21	0.58
ES25	0.01	0.36	-0.21	0.88
ES6	0.11	0.31	0.02	0.93
ES5	0.02	0.31	-0.01	0.91
ES23	-0.14	0.21	0.19	0.83
ES34	-0.12	0.18	**0.68**	0.37
ES33	-0.17	0.05	**0.65**	0.49
ES38	-0.07	0.04	**0.64**	0.55
ES45	-0.09	0.21	**0.59**	0.49
ES37	0.08	-0.10	**0.57**	0.71
ES16	0.39	-0.39	**0.40**	0.52
ES21	0.30	-0.06	0.38	0.80
ES9	0.15	-0.00	0.26	0.93

*Note*. Items highlighted in yellow, violet, and green have been selected for a concise version of the ES subscales: *Harmonious Body, Body-for-Others*, and *Disharmonious Body*, respectively.

### Exploratory Factor Analysis of the 13-item Version

Oblimin rotation was utilized in combination with principal-axis factoring extraction to conduct EFA on the 13-item version with data from Sample 2. A 3-factor model was derived with very good fit (RMSEA = .05, 90% CI [.03; .06], TLI = .97, *χ^2^*(42) = 83.7, *p* < .001). All items except item 8, “I ignore my body's needs (for food, rest, or movement)” loaded on expected factors as identified in previous EFA analyses using Sample 1. Furthermore, this item loaded lower than .4 on the factor. Following discussions within our research group and with a group of adolescents, this item was excluded from the scale. Factor loadings are presented in Table A3, together with a preliminary English translation of items in the ES-12 (the original Swedish items are seen in Table A5).

**Table A3:** Exploratory Factor Loadings for the 13-Item Version of the ES-12 (Sample 2).

Item	Factor			Uniqueness
	1	2	3	
10. I enjoy having the body I have.	**0.93**	-0.06	0.09	0.18
1. I am friends with my body.	**0.88**	0.01	-0.08	0.14	
13. I feel at home in my body.	**0.77**	0.03	-0.08	0.22	
22. My body reflects who I feel I am inside.	**0.62**	0.13	0.03	0.70	
12. I care more about how the body feels than how it looks.	**0.42**	-0.38	0.04	0.56	
8. I ignore my body's needs (for food, rest, or movement).	-0.34	0.12	0.27	0.61	
45. It is important to me what other people think about my appearance and physical characteristics.	-0.01	**0.98**	-0.01	0.04	
38. It is important to me that others do not think I am physically weak.	0.09	**0.67**	-0.01	0.61	
34. I think about how my body looks to others.	-0.07	**0.65**	0.13	0.44	
28. It happens that my body feels completely foreign to me.	0.04	0.01	**0.84**	0.33	
19. I can feel separated and disconnected from my body.	-0.08	0.03	**0.67**	0.43	
4. It is unpleasant to feel what it feels like in my body.	-0.28	-0.00	**0.44**	0.56	
24. My body prevents me from doing what I want (such as playing sports or hanging out with friends).	-0.08	0.11	**0.40**	0.74	

### Internal consistency of the validation variables

The internal consistency coefficients (Cronbach’s α) for the variables used for evaluation of convergent and discriminant validity of the ES-12 across samples are shown in Table A4.

**Table A4:** Internal Consistency Coefficients (Cronbach’s α) for Variables Used for Evaluation of Convergent and Discriminant Validity of the ES-12 Across Samples.

Scale/subscale	Sample 2 (*n* = 236-237)	Sample 3 (*n* = 281-283)	Combined (*n* = 517-520)
**BESAA**	.94	.95	.94
**RSES**	.91	.94	.92
**Life satisfaction**	.88	.91	.90
**MAIA-2**			
Noticing	.70	.77	.74
Non-distracting	.78	.79	.79
Self-regulation	.82	.81	.81
Non-worrying	.56	.57	.56
Attention regulation	.80	.83	.82
Emotional awareness	.75	.82	.80
Body listening	.85	.78	.82
Trusting	.88	.89	.89
**SDQ-s**			
Hyperactivity/inattention	.78	.78	.78
Conduct problems	.63	.55	.56
Emotional symptoms	.77	.75	.74
Peer problems	.48	.63	.59
**RCADS-25**			
Anxiety	.87	.87	.86
Depression	.86	.89	.88
**RIBED-8**	.88	.88	.88
**DSHI-9r**	.90	.88	.88

*Note.* BESAA = Body Esteem Scale for Adolescents and Adults – Appearance subscale; MAIA-2 = Multidimensional Assessment of Interoceptive Awareness 2; RSES = Rosenberg’s Self-Esteem Scale; SDQ-s = Strength and Difficulties Questionnaire – self-report version; RCADS-25 = Revised Child Anxiety and Depression Scale - shortened version; RIBED-8 = Risk Behaviour related to Eating Disorders; DSHI-9r – Deliberate Self-Harm Inventory – 9 item

### The ES-12 with subscales and items, in Swedish original, and English translation

Table A5 shows the final E2-12 with subscales and items, in Swedish original as used in the present study, and in a preliminary English translation.

**Table A5:** The Swedish Version and an English Translation of the items in the 12-item Embodiment Scale (ES-12).

In Swedish	English translation
**Harmonisk kropp**	**Harmonious body**
1. Jag är vän med min kropp.	1. I am friends with my body.
10. Jag njuter av att ha den kropp jag har.	10. I enjoy having the body I have.
12. Jag bryr mig mer om hur kroppen känns än hur den ser ut.	12. I care more about how the body feels than how it looks.
13. Jag känner mig hemma i min kropp.	13. I feel at home in my body.
22. Min kropp återspeglar den jag känner att jag är inombords.	22. My body reflects who I feel I am inside.
**Disharmonisk kropp**	**Disharmonious body**
4. Det är obehagligt att känna efter hur det känns i kroppen.	4. It is unpleasant to feel what it feels like in my body.
19. Jag kan känna mig åtskild och avstängd från min kropp.	19. I can feel separated and disconnected from my body.
24. Min kropp hindrar mig från att göra det jag vill (som t.ex. att sporta, eller att umgås med kompisar).	24. My body prevents me from doing what I want (such as playing sports or hanging out with friends).
28. Det händer att min kropp känns helt främmande för mig.	28. It happens that my body feels completely foreign to me.
**Kropp-för andra**	**Body-for-others**
34. Jag tänker på hur min kropp ser ut för andra.	34. I think about how my body looks to others.
38. Det är viktigt för mig att andra inte tycker att jag är fysiskt svag.	38. It is important to me that others do not think I am physically weak.
45. Det är viktigt för mig vad andra tänker om mitt utseende och mina fysiska egenskaper.	45. It is important to me what other people think about my appearance and physical characteristics.
